# Supplementary material for: Antioxidant Activity and GC-MS Profile of Cardamom (Elettaria cardamomum) Essential Oil Obtained by a Combined Extraction Method—Instant Controlled Pressure Drop Technology Coupled with Sonication
Source: Molecules. 2023 Jan 21;28(3):1093. doi: 10.3390/molecules28031093 (PMC9920946; doi:10.3390/molecules28031093)
Supplement: Supplementary file 1 [file molecules-28-01093-s001.zip › molecules-2114930-supplementary.pdf]

Supplementary materials

Antioxidant Activity and GC-MS Profile of Cardamom (*Elettaria cardamomum)* Essential Oil Obtained by a Combined Extraction Method—Instant Controlled Pressure Drop Technology Coupled with Sonication

a) TAC

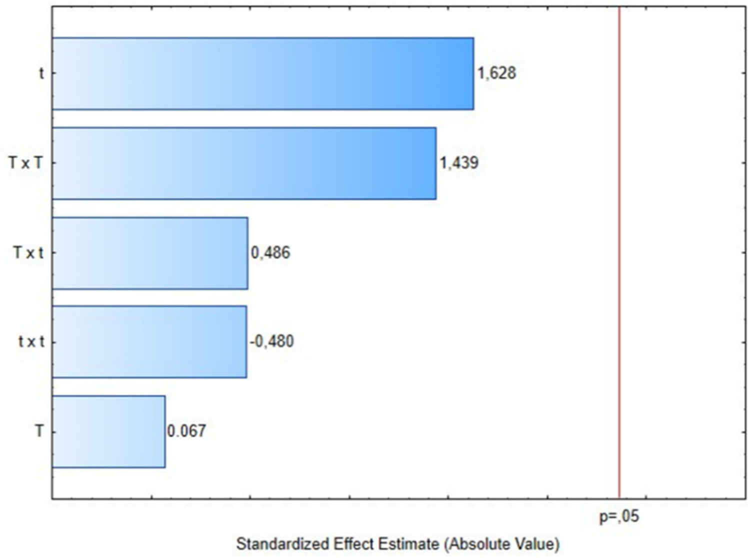

b) GO

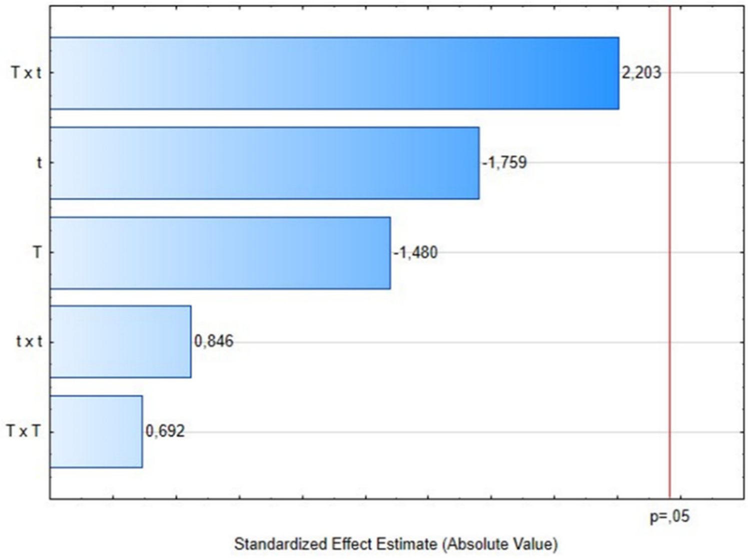

c) OCAD

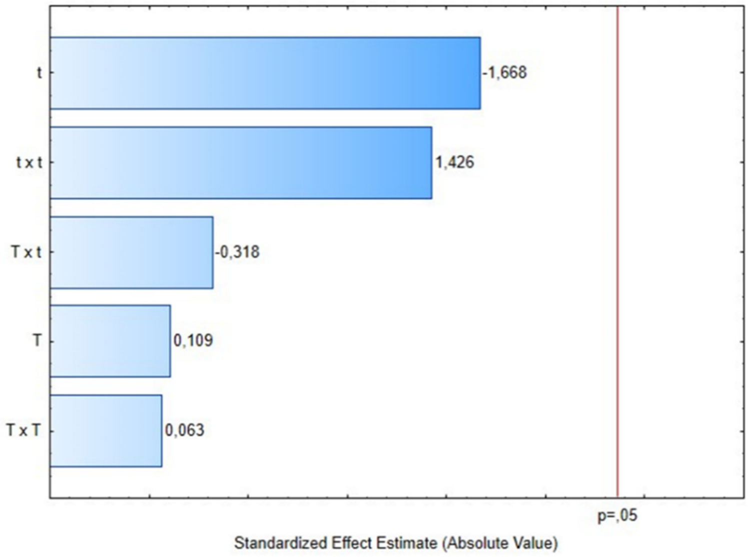

d) HEXAD

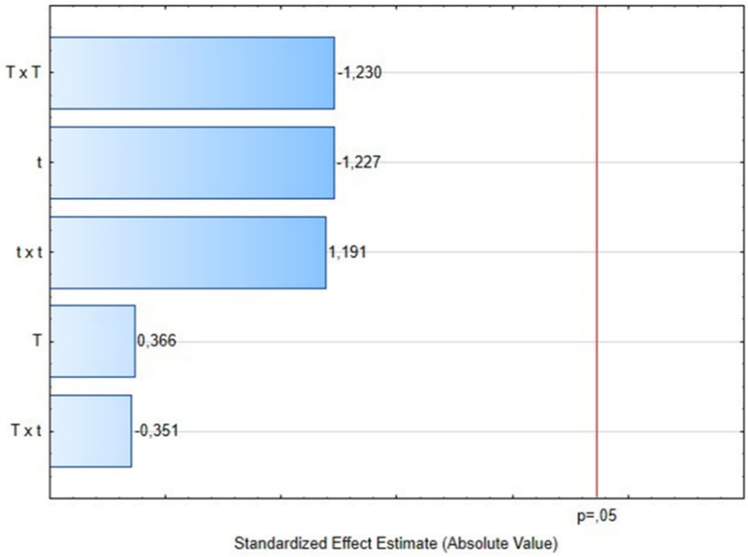

Figure S1. Pareto charts evaluating the effect of steam processing temperature "T" (°C) and thermal processing time "t" (s) on some EO compounds: a. TAC (α-Terpinyl acetate), b. GO (Geranyl oleate), c. OCAD (9,12-Octadecadienoic acid (Z,Z)-), and d. HEXAD (n-Hexadecanoic acid).

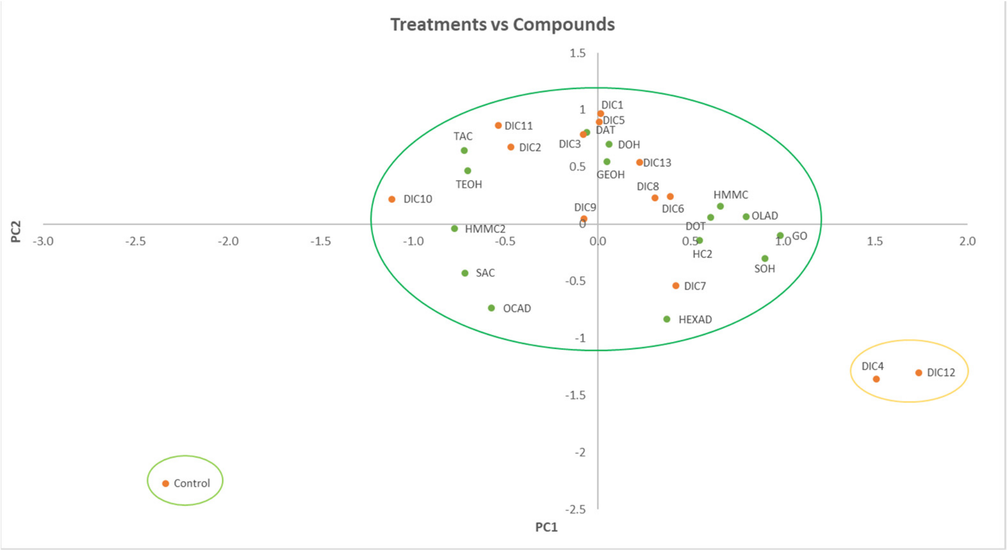

Figure S2. Biplot of variables and treatments in the factorial design. The correlation between the compounds found in the treatments can be observed in the figure. Each point indicates a compound, and these were grouped into three large data sets according to their treatment similarities.

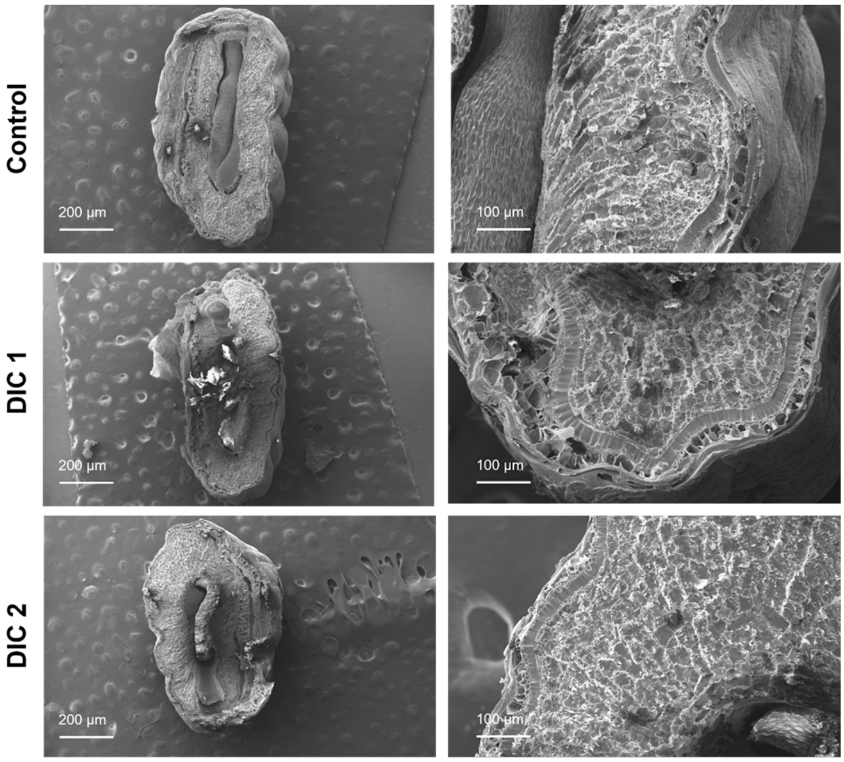

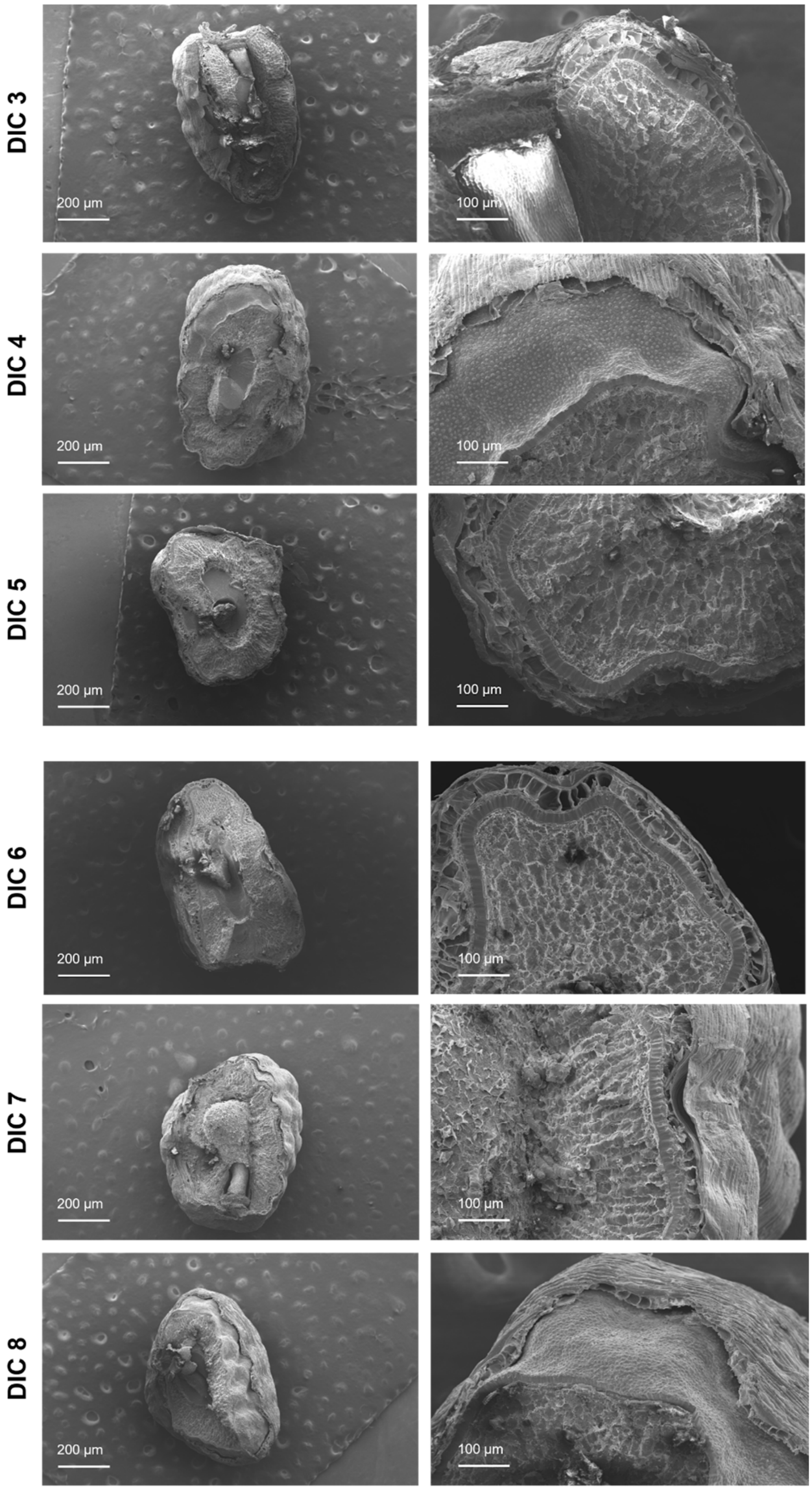
DIC 3
200 µm
100 µm
DIC 4
200 µm
100 µm
DIC 5
200 µm
100 µm
DIC 6
200 µm
100 µm
DIC 7
200 µm
100 µm
DIC 8
200 µm
100 µm

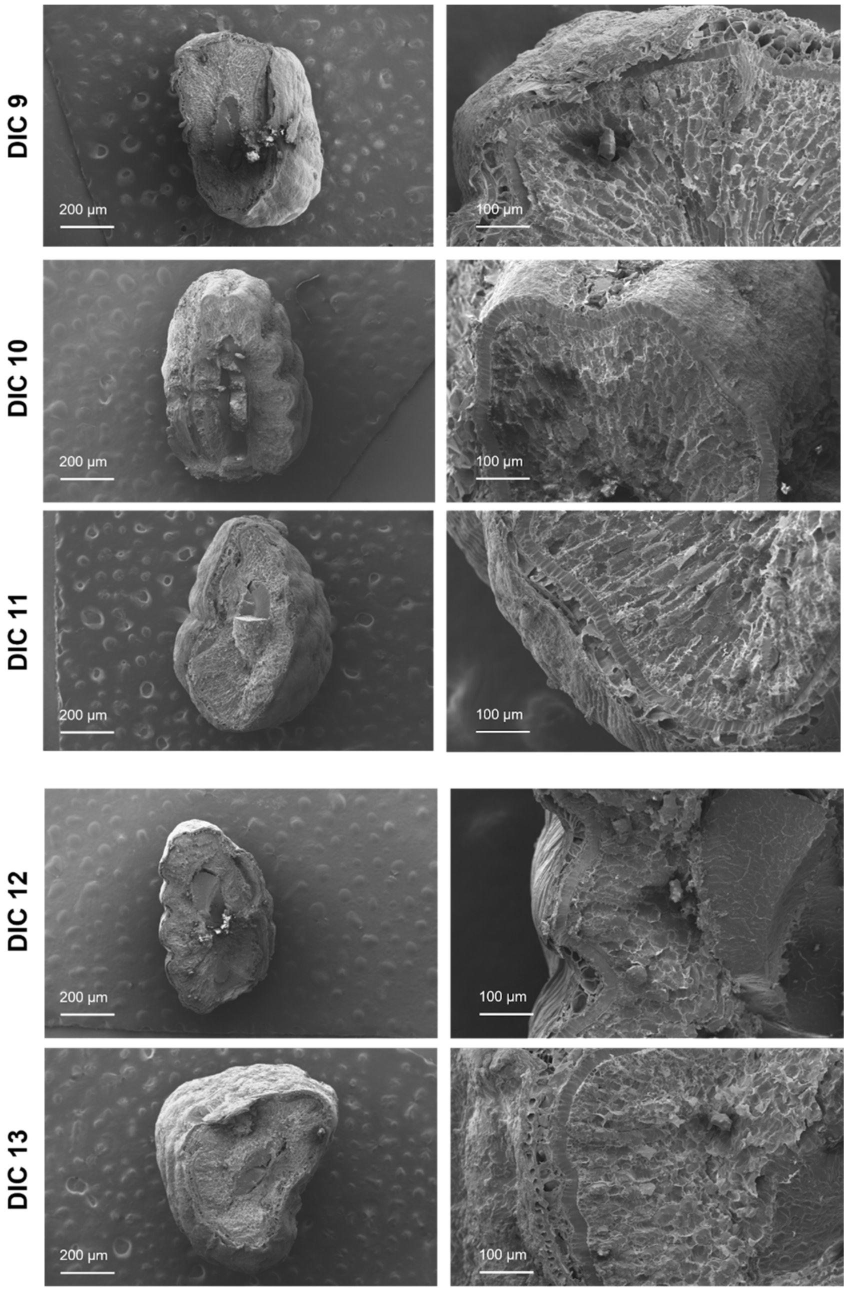

Figure S3. SEM micrographs of cardamom seeds (through a cross-section cut) treated by DIC and the control (untreated). The bars show two scales according to the magnitude employed (200 μm for 22X and 100 μm for 110X)

Qualitative Report

File: C:\TurboMass\Biotec.PRO\Data\Control.raw
Acquired: 09-Feb-22 10:34:12 PM
Printed: 16-Feb-22 12:06 PM
Description:
GC/MS Method: GC: MS: Vainilla.EXP
Page 1 of 1
Sample ID: Control
Vial Number: 15

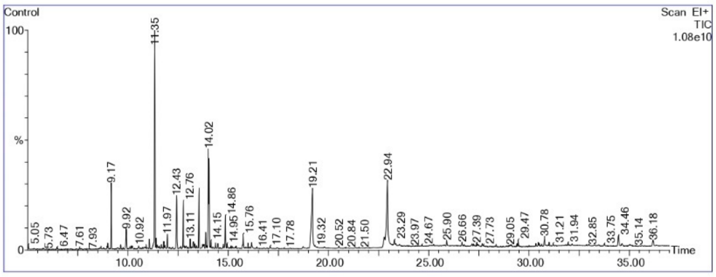

#	RT	Scan	Height	Area	Area %	Norm %
1	9.174	626	3,262,759,680	83,730,504.0	3.356	20.96
2	9.921	738	1,000,222,016	36,155,156.0	1.449	9.05
3	11.348	952	10,589,813,760	399,411,360.0	16.007	100.00
4	11.968	1045	712,936,512	22,715,798.0	0.910	5.69
5	12.435	1115	2,615,351,296	95,269,896.0	3.818	23.85
6	12.762	1164	2,314,913,280	58,039,848.0	2.326	14.53
7	13.548	1282	2,967,782,144	77,607,016.0	3.110	19.43
8	13.889	1333	777,160,448	19,114,378.0	0.766	4.79
9	14.015	1352	4,687,321,088	103,216,616.0	4.137	25.84
10	14.049	1357	4,274,066,688	115,667,864.0	4.636	28.96
11	14.862	1479	1,608,838,912	42,614,788.0	1.708	10.67
12	15.756	1613	696,175,680	22,188,662.0	0.889	5.56
13	19.210	2131	2,966,042,624	267,436,240.0	10.718	66.96
14	22.791	2668	513,013,504	43,587,184.0	1.747	10.91
15	22.945	2691	3,314,389,760	307,073,472.0	12.306	76.88
16	23.291	2743	362,824,416	22,471,224.0	0.901	5.63
17	23.545	2781	128,149,792	17,645,984.0	0.707	4.42
18	30.780	3866	480,700,640	21,878,244.0	0.877	5.48
19	34.461	4418	524,345,984	27,947,136.0	1.120	7.00
20	36.182	4676	293,416,928	18,241,404.0	0.731	4.57

Inst() ACQUISITION PARAMETERS
, Inj=°C, Volume=0 µL, Split=:1, Carrier Gas=, Solvent Delay=5.00 min, Transfer Temp=210°C, Source Temp=210°C, Scan: 30 to 550Da, Column

Figure S4. Chromatograms and Quality Report retrieved from de GC-MS analysis with the peaks corresponding to the most abundant compounds for the control – untreated seeds + UAE – (**Table 1**).

Qualitative Report

File: C:\TurboMass\Biotec.PRO\Data\Mta 51.raw
Acquired: 09-Feb-22 11:34:13 AM
Description:
GC/MS Method: GC: MS: Vainilla.EXP
Sample ID: Mta 1

Printed: 16-Feb-22 09:59 AM
Page 1 of 1
Vial Number: 2

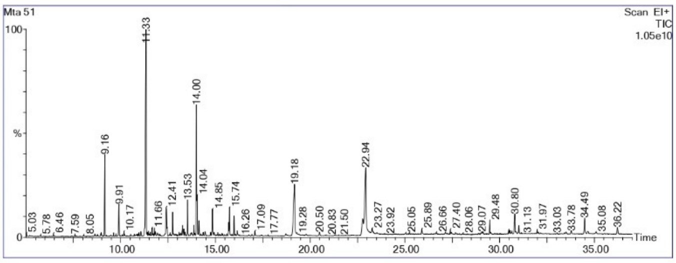

#	RT	Scan	Height	Area	Area %	Norm %
1	9.161	624	4,108,384,256	107,943,912.0	4.064	21.21
2	9.914	737	1,597,205,888	51,860,852.0	1.952	10.19
3	11.334	950	10,386,978,816	508,924,736.0	19.160	100.00
4	12.408	1111	1,443,118,592	83,555,480.0	3.146	16.42
5	12.748	1162	1,183,466,368	32,855,910.0	1.237	6.46
6	13.528	1279	1,808,343,040	47,953,612.0	1.805	9.42
7	14.002	1350	6,537,811,968	224,442,864.0	8.450	44.10
8	14.135	1370	716,143,488	17,810,268.0	0.671	3.50
9	14.849	1477	1,318,520,448	38,696,056.0	1.457	7.60
10	15.696	1604	674,709,248	16,518,081.0	0.622	3.25
11	15.742	1611	1,410,956,160	37,496,004.0	1.412	7.37
12	15.983	1647	990,397,952	24,418,912.0	0.919	4.80
13	19.177	2126	2,588,513,024	227,816,128.0	8.577	44.76
14	22.785	2667	827,691,392	72,192,808.0	2.718	14.19
15	22.938	2690	3,397,783,040	327,129,184.0	12.316	64.28
16	23.271	2740	324,585,568	31,470,616.0	1.185	6.18
17	29.480	3671	690,319,808	19,756,496.0	0.744	3.88
18	30.800	3869	993,088,832	38,770,260.0	1.460	7.62
19	34.495	4423	805,139,584	41,885,396.0	1.577	8.23
20	36.215	4681	354,498,016	22,846,418.0	0.860	4.49

Inst() ACQUISITION PARAMETERS
, Inj=°C, Volume=0 µL, Split=:1, Carrier Gas=, Solvent Delay=5.00 min, Transfer Temp=210°C, Source Temp=210°C, Scan: 30 to 550Da, Column

Figure S5. Chromatograms and Quality Report retrieved from de GC-MS analysis with the peaks corresponding to the most abundant compounds for the seeds under the treatment 1 + UAE (**Table 1**).

Qualitative Report

File: C:\TurboMass\Biotec.PRO\Data\Mta 52.raw
Acquired: 09-Feb-22 12:15:25 PM
Printed: 16-Feb-22 10:36 AM
Description:
GC/MS Method: GC: MS: Vainilla.EXP
Page 1 of 1
Sample ID: Mta 2
Vial Number: 3

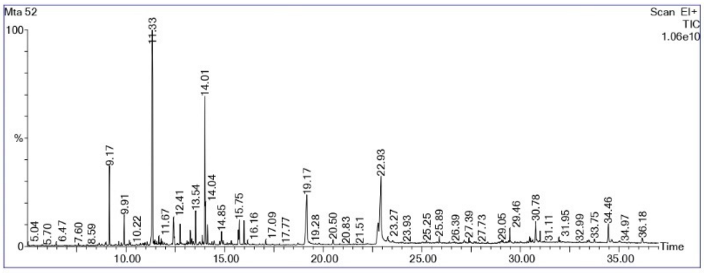

#	RT	Scan	Height	Area	Area %	Norm %
1	9.167	625	3,893,965,312	100,912,600.0	3.829	18.88
2	9.914	737	1,501,927,936	48,228,548.0	1.830	9.02
3	11.334	950	10,512,866,304	534,612,704.0	20.287	100.00
4	12.408	1111	1,339,252,992	63,845,532.0	2.423	11.94
5	12.748	1162	1,025,669,952	25,885,174.0	0.982	4.84
6	13.268	1240	670,522,752	17,899,406.0	0.679	3.35
7	13.535	1280	1,673,518,080	43,905,876.0	1.666	8.21
8	14.009	1351	7,237,666,304	239,799,072.0	9.100	44.85
9	14.142	1371	961,236,416	24,547,332.0	0.932	4.59
10	14.849	1477	640,084,352	21,117,700.0	0.801	3.95
11	15.702	1605	723,878,464	19,294,448.0	0.732	3.61
12	15.749	1612	1,214,678,400	32,480,412.0	1.233	6.08
13	15.989	1648	1,169,107,072	27,580,082.0	1.047	5.16
14	19.170	2125	2,429,329,664	210,713,984.0	7.996	39.41
15	22.785	2667	1,027,949,184	87,613,752.0	3.325	16.39
16	22.931	2689	3,313,788,416	308,604,416.0	11.711	57.72
17	23.271	2740	315,925,376	35,399,868.0	1.343	6.62
18	29.460	3668	705,539,456	20,318,430.0	0.771	3.80
19	30.780	3866	1,013,440,320	38,606,776.0	1.465	7.22
20	34.461	4418	917,220,416	48,306,568.0	1.833	9.04

Inst() ACQUISITION PARAMETERS
, Inj=°C, Volume=0 µL, Split=:1, Carrier Gas=, Solvent Delay=5.00 min, Transfer Temp=210°C, Source Temp=210°C, Scan: 30 to 550Da, Column

Figure S6. Chromatograms and Quality Report retrieved from de GC-MS analysis with the peaks corresponding to the most abundant compounds for the seeds under the treatment 2 + UAE (**Table 1**).

Qualitative Report

File:	C:\TurboMass\Biotec.PRO\Data\Mta 53.raw		
Acquired:	09-Feb-22 12:56:35 PM		Printed: 16-Feb-22 10:45 AM
Description:			
GC/MS Method:	GC:	MS: Vainilla.EXP	Page 1 of 1
Sample ID:	Mta 3		Vial Number: 4

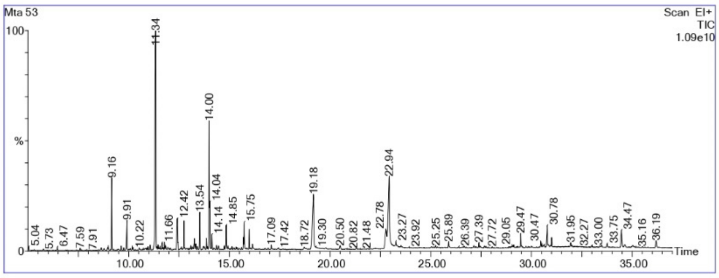

#	RT	Scan	Height	Area	Area %	Norm %
1	9.160	624	3,587,598,848	96,761,064.0	3.613	19.04
2	9.914	737	1,486,116,224	46,646,220.0	1.742	9.18
3	11.341	951	10,842,103,808	508,278,816.0	18.981	100.00
4	12.421	1113	1,547,108,992	74,353,784.0	2.777	14.63
5	12.748	1162	1,421,965,440	37,218,820.0	1.390	7.32
6	13.535	1280	1,856,688,640	50,231,948.0	1.876	9.88
7	14.002	1350	6,352,412,672	231,520,016.0	8.646	45.55
8	14.135	1370	726,955,968	18,211,122.0	0.680	3.58
9	14.849	1477	1,176,958,336	35,077,004.0	1.310	6.90
10	15.696	1604	606,307,008	17,989,166.0	0.672	3.54
11	15.749	1612	1,339,262,464	34,813,804.0	1.300	6.85
12	15.989	1648	979,865,280	23,747,772.0	0.887	4.67
13	19.177	2126	2,718,254,848	232,633,296.0	8.687	45.77
14	22.778	2666	941,102,592	82,024,136.0	3.063	16.14
15	22.938	2690	3,580,773,632	329,059,456.0	12.288	64.74
16	23.271	2740	347,414,304	39,996,904.0	1.494	7.87
17	29.467	3669	698,828,160	19,325,828.0	0.722	3.80
18	30.780	3866	1,044,353,984	39,848,412.0	1.488	7.84
19	34.468	4419	831,576,768	43,373,612.0	1.620	8.53
20	36.189	4677	349,271,776	21,291,832.0	0.795	4.19

Inst() ACQUISITION PARAMETERS
, Inj=°C, Volume=0 µL, Split=:1, Carrier Gas=, Solvent Delay=5.00 min, Transfer Temp=210°C, Source Temp=210°C, Scan: 30 to 550Da, Column

Figure S7. Chromatograms and Quality Report retrieved from de GC-MS analysis with the peaks corresponding to the most abundant compounds for the seeds under the treatment 3 + UAE (**Table 1**).

Qualitative Report

File: C:\TurboMass\BIOTEC.PRO\Data\Mta 54.raw
Acquired: 09-Feb-22 01:37:47 PM
Description:
GC/MS Method: GC: MS: Vainilla.EXP
Sample ID: Mta 4

Printed: 19-Nov-22 10:38 AM
Page 1 of 2
Vial Number: 5

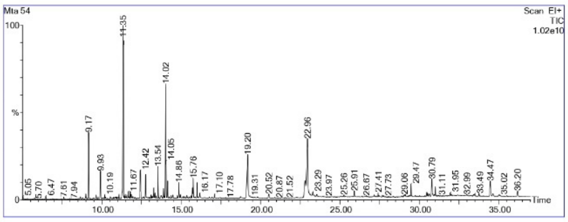

#	RT	Scan	Height	Area	Area %	Norm %
1	9.174	626	3,894,785,024	100,132,504.0	5.391	20.46
2	9.927	739	1,630,676,864	50,496,452.0	2.719	10.32
3	11.348	952	10,019,212,288	489,496,160.0	26.354	100.00
4	12.422	1113	1,636,689,792	79,880,312.0	4.301	16.32
5	12.762	1164	1,348,303,616	31,698,968.0	1.707	6.48
6	13.282	1242	587,512,448	17,688,570.0	0.952	3.61
7	13.542	1281	1,879,521,280	50,034,836.0	2.694	10.22
8	13.889	1333	545,094,784	12,886,680.0	0.694	2.63
9	14.015	1352	6,686,499,328	236,665,120.0	12.742	48.35
10	14.149	1372	998,018,624	23,089,572.0	1.243	4.72
11	14.856	1478	914,287,744	28,653,318.0	1.543	5.85
12	15.709	1606	581,965,056	17,299,618.0	0.931	3.53
13	15.756	1613	1,131,884,288	28,027,582.0	1.509	5.73
14	15.996	1649	899,776,384	21,576,348.0	1.162	4.41
15	19.197	2129	2,536,468,992	209,683,152.0	11.289	42.84
16	22.805	2670	906,183,040	74,245,520.0	3.997	15.17
17	22.958	2693	3,333,453,568	271,612,320.0	14.624	55.49
18	29.474	3670	689,341,568	17,373,856.0	0.935	3.55
19	30.794	3868	936,628,672	35,281,408.0	1.900	7.21
20	31.014	3901	527,924,544	14,617,690.0	0.787	2.99

Inst() ACQUISITION PARAMETERS
, Inj=°C, Volume=0 µL, Split=:1, Carrier Gas=, Solvent Delay=5.00 min, Transfer Temp=210°C, Source Temp=210°C, Scan: 30 to 550Da, Column

Figure S8. Chromatograms and Quality Report retrieved from de GC-MS analysis with the peaks corresponding to the most abundant compounds for the seeds under the treatment 4 + UAE (**Table 1**).

Qualitative Report

File: C:\TurboMass\Biotec.PRO\Data\Mta 55.raw
Acquired: 09-Feb-22 02:19:00 PM   Printed: 16-Feb-22 11:06 AM
Description:
GC/MS Method: GC: MS: Vainilla.EXP   Page 1 of 1
Sample ID: Mta 5   Vial Number: 6

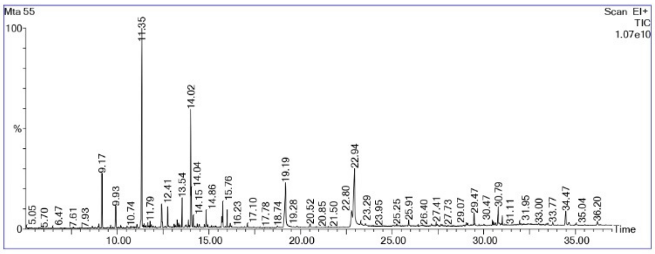

#	RT	Scan	Height	Area	Area %	Norm %
1	9.174	626	2,916,642,560	76,154,784.0	3.225	17.23
2	9.927	739	1,177,870,336	36,999,212.0	1.567	8.37
3	11.348	952	10,590,707,712	441,987,936.0	18.716	100.00
4	12.435	1115	1,254,280,704	63,292,880.0	2.680	14.32
5	12.755	1163	1,103,880,960	28,662,980.0	1.214	6.49
6	13.542	1281	1,588,264,576	40,884,844.0	1.731	9.25
7	14.015	1352	6,248,120,832	213,247,072.0	9.030	48.25
8	14.149	1372	619,890,112	15,562,381.0	0.659	3.52
9	14.856	1478	900,590,976	27,531,430.0	1.166	6.23
10	15.709	1606	575,281,024	15,783,652.0	0.668	3.57
11	15.756	1613	1,346,192,000	34,282,076.0	1.452	7.76
12	15.996	1649	942,634,496	22,073,040.0	0.935	4.99
13	19.190	2128	2,385,511,680	203,842,032.0	8.632	46.12
14	22.805	2670	820,304,768	68,760,800.0	2.912	15.56
15	22.945	2691	3,101,534,976	283,878,176.0	12.021	64.23
16	23.291	2743	301,802,016	33,580,548.0	1.422	7.60
17	23.552	2782	158,631,456	17,150,216.0	0.726	3.88
18	29.473	3670	672,556,352	18,547,156.0	0.785	4.20
19	30.794	3868	977,344,512	38,123,276.0	1.614	8.63
20	34.475	4420	740,338,688	38,878,224.0	1.646	8.80

Inst() ACQUISITION PARAMETERS
, Inj=°C, Volume=0 µL, Split=:1, Carrier Gas=, Solvent Delay=5.00 min, Transfer Temp=210°C, Source Temp=210°C, Scan: 30 to 550Da, Column

Figure S9. Chromatograms and Quality Report retrieved from de GC-MS analysis with the peaks corresponding to the most abundant compounds for the seeds under the treatment 5 + UAE (**Table 1**).

Qualitative Report

File: C:\TurboMass\Biotec.PRO\Data\Mta 56.raw
Acquired: 09-Feb-22 03:41:31 PM
Description:
GC/MS Method: GC: MS: Vainilla.EXP
Sample ID: Mta 6

Printed: 16-Feb-22 11:18 AM
Page 1 of 1
Vial Number: 7

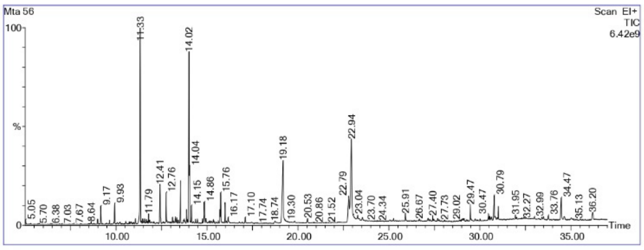

#	RT	Scan	Height	Area	Area %	Norm %
1	9.174	626	585,759,936	15,441,020.0	0.889	6.39
2	9.927	739	663,523,776	16,168,082.0	0.931	6.69
3	11.334	950	6,373,322,752	193,710,224.0	11.158	80.17
4	12.415	1112	1,277,262,080	58,515,844.0	3.371	24.22
5	12.762	1164	965,934,464	23,781,180.0	1.370	9.84
6	13.542	1281	1,384,022,016	36,180,600.0	2.084	14.97
7	14.015	1352	5,558,594,560	186,674,720.0	10.753	77.26
8	14.149	1372	554,883,392	12,994,188.0	0.749	5.38
9	14.856	1478	678,258,240	21,095,416.0	1.215	8.73
10	15.756	1613	983,798,976	26,534,898.0	1.529	10.98
11	15.996	1649	664,953,216	15,917,872.0	0.917	6.59
12	19.184	2127	2,043,234,944	163,145,952.0	9.398	67.52
13	22.791	2668	840,053,568	65,403,168.0	3.767	27.07
14	22.945	2691	2,714,172,672	241,627,232.0	13.919	100.00
15	23.285	2742	230,378,128	15,465,009.0	0.891	6.40
16	23.545	2781	141,135,952	15,394,645.0	0.887	6.37
17	29.473	3670	546,081,472	15,486,160.0	0.892	6.41
18	30.787	3867	783,534,528	30,416,906.0	1.752	12.59
19	34.469	4419	708,999,424	36,201,452.0	2.085	14.98
20	36.196	4678	246,098,976	15,523,875.0	0.894	6.42

Inst() ACQUISITION PARAMETERS
, Inj=°C, Volume=0 µL, Split=:1, Carrier Gas=, Solvent Delay=5.00 min, Transfer Temp=210°C, Source Temp=210°C, Scan: 30 to 550Da, Column

Figure S10. Chromatograms and Quality Report retrieved from de GC-MS analysis with the peaks corresponding to the most abundant compounds for the seeds under the treatment 6 + UAE (**Table 1**).

Qualitative Report

File: C:\TurboMass\Biotec.PRO\Data\Mta 57.raw
Acquired: 09-Feb-22 04:22:44 PM
Printed: 16-Feb-22 11:27 AM
Description:
GC/MS Method: GC: MS: Vainilla.EXP
Page 1 of 1
Sample ID: Mta 7
Vial Number: 8

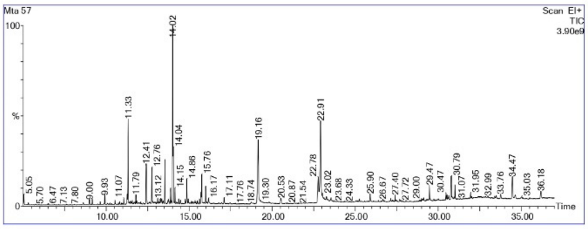

#	RT	Scan	Height	Area	Area %	Norm %
1	11.274	941	216,715,632	8,623,581.0	0.813	6.71
2	11.328	949	1,835,023,744	48,069,448.0	4.531	37.42
3	12.415	1112	868,347,072	40,356,292.0	3.804	31.42
4	12.762	1164	764,732,608	17,606,372.0	1.660	13.71
5	13.542	1281	948,586,112	23,372,944.0	2.203	18.20
6	14.015	1352	3,833,227,008	121,848,928.0	11.486	94.87
7	14.856	1478	522,787,680	16,457,391.0	1.551	12.81
8	15.762	1614	617,605,184	15,462,417.0	1.458	12.04
9	16.003	1650	376,534,976	9,095,519.0	0.857	7.08
10	19.164	2124	1,377,215,872	98,652,064.0	9.299	76.81
11	22.778	2666	553,734,144	35,459,744.0	3.342	27.61
12	22.911	2686	1,772,066,944	128,444,472.0	12.107	100.00
13	23.025	2703	196,136,640	20,714,838.0	1.953	16.13
14	23.278	2741	128,013,960	10,799,630.0	1.018	8.41
15	23.545	2781	102,990,360	12,458,204.0	1.174	9.70
16	29.473	3670	340,647,424	10,848,017.0	1.023	8.45
17	30.787	3867	495,215,552	19,025,824.0	1.793	14.81
18	31.007	3900	303,180,384	8,930,631.0	0.842	6.95
19	34.468	4419	466,193,120	23,484,274.0	2.214	18.28
20	36.182	4676	154,291,664	9,899,532.0	0.933	7.71

Inst() ACQUISITION PARAMETERS
, Inj=°C, Volume=0 µL, Split=:1, Carrier Gas=, Solvent Delay=5.00 min, Transfer Temp=210°C, Source Temp=210°C, Scan: 30 to 550Da, Column

Figure S11. Chromatograms and Quality Report retrieved from de GC-MS analysis with the peaks corresponding to the most abundant compounds for the seeds under the treatment 7 + UAE (**Table 1**).

Qualitative Report

File: C:\TurboMass\Biotec.PRO\Data\Mta 58.raw
Acquired: 09-Feb-22 05:03:59 PM   Printed: 16-Feb-22 11:33 AM
Description:
GC/MS Method: GC: MS: Vainilla.EXP   Page 1 of 1
Sample ID: Mta 8   Vial Number: 9

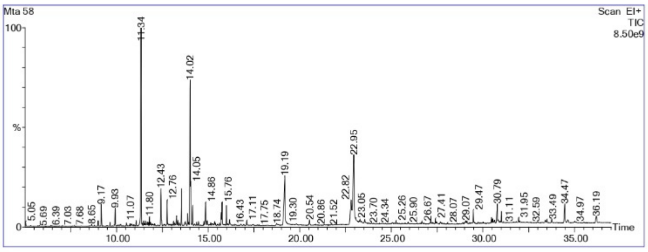

#	RT	Scan	Height	Area	Area %	Norm %
1	9.174	626	929,241,600	23,392,624.0	1.192	8.63
2	9.927	739	767,498,880	19,450,048.0	0.991	7.17
3	11.341	951	8,443,316,736	271,087,488.0	13.814	100.00
4	12.428	1114	1,588,727,296	70,013,304.0	3.568	25.83
5	12.762	1164	1,060,214,592	26,700,870.0	1.361	9.85
6	13.548	1282	1,569,722,240	40,085,304.0	2.043	14.79
7	14.022	1353	6,177,482,240	205,931,536.0	10.494	75.96
8	14.155	1373	852,354,688	20,692,446.0	1.054	7.63
9	14.862	1479	961,266,624	29,962,434.0	1.527	11.05
10	15.716	1607	542,846,400	15,470,372.0	0.788	5.71
11	15.762	1614	972,915,136	25,193,484.0	1.284	9.29
12	16.003	1650	846,463,104	19,972,968.0	1.018	7.37
13	19.190	2128	2,112,752,640	171,064,368.0	8.717	63.10
14	22.805	2670	988,369,088	80,997,528.0	4.128	29.88
15	22.951	2692	2,931,291,392	250,872,464.0	12.784	92.54
16	29.473	3670	560,442,112	15,326,306.0	0.781	5.65
17	30.787	3867	807,616,768	33,324,610.0	1.698	12.29
18	31.007	3900	472,714,144	13,725,368.0	0.699	5.06
19	34.468	4419	799,091,968	42,235,096.0	2.152	15.58
20	36.189	4677	291,764,192	18,786,800.0	0.957	6.93

Inst() ACQUISITION PARAMETERS
, Inj=°C, Volume=0 µL, Split=:1, Carrier Gas=, Solvent Delay=5.00 min, Transfer Temp=210°C, Source Temp=210°C, Scan: 30 to 550Da, Column

Figure S12. Chromatograms and Quality Report retrieved from de GC-MS analysis with the peaks corresponding to the most abundant compounds for the seeds under the treatment 8 + UAE (**Table 1**).

Qualitative Report

File: C:\TurboMass\Biotec.PRO\Data\Mta 59.raw
Acquired: 09-Feb-22 05:45:14 PM
Printed: 17-Mar-22 04:34 PM
Description:
GC/MS Method: GC: MS: Vainilla.EXP
Page 1 of 1
Sample ID: Mta 9
Vial Number: 10

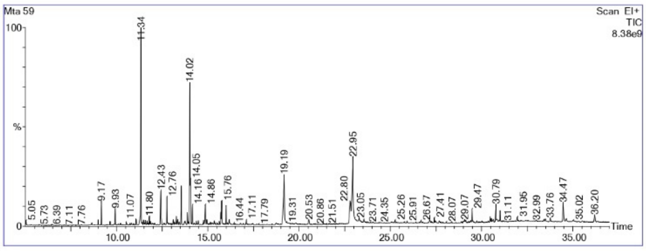

#	RT	Scan	Height	Area	Area %	Norm %
1	9.174	626	1,023,921,728	25,206,506.0	1.305	9.03
2	9.934	740	702,504,192	18,704,662.0	0.969	6.70
3	11.341	951	8,322,854,912	279,084,320.0	14.451	100.00
4	12.435	1115	1,485,233,152	68,999,952.0	3.573	24.72
5	12.762	1164	1,160,168,448	30,066,022.0	1.557	10.77
6	13.549	1282	1,640,787,200	41,785,244.0	2.164	14.97
7	13.889	1333	501,304,864	13,114,424.0	0.679	4.70
8	14.022	1353	5,971,261,440	201,016,784.0	10.409	72.03
9	14.155	1373	813,753,600	18,985,488.0	0.983	6.80
10	14.862	1479	826,100,928	25,902,234.0	1.341	9.28
11	15.716	1607	518,547,712	14,320,499.0	0.742	5.13
12	15.763	1614	985,688,768	25,897,718.0	1.341	9.28
13	16.003	1650	795,536,704	18,932,720.0	0.980	6.78
14	19.190	2128	2,102,420,352	168,707,888.0	8.736	60.45
15	22.805	2670	885,424,512	69,305,656.0	3.589	24.83
16	22.951	2692	2,793,865,728	237,225,152.0	12.284	85.00
17	29.473	3670	566,194,048	16,100,492.0	0.834	5.77
18	30.787	3867	743,924,352	30,857,108.0	1.598	11.06
19	34.468	4419	815,613,504	40,787,168.0	2.112	14.61
20	36.196	4678	316,292,704	19,350,474.0	1.002	6.93

Inst() ACQUISITION PARAMETERS
, Inj=°C, Volume=0 µL, Split=:1, Carrier Gas=, Solvent Delay=5.00 min, Transfer Temp=210°C, Source Temp=210°C, Scan: 30 to 550Da, Column

Figure S13. Chromatograms and Quality Report retrieved from de GC-MS analysis with the peaks corresponding to the most abundant compounds for the seeds under the treatment 9 + UAE (**Table 1**).

Qualitative Report

File: C:\TurboMass\Biotec.PRO\Data\Mta 60.raw
Acquired: 09-Feb-22 06:26:29 PM
Printed: 16-Feb-22 11:41 AM
Description:
GC/MS Method: GC: MS: Vainilla.EXP
Page 1 of 1
Sample ID: Mta 10
Vial Number: 11

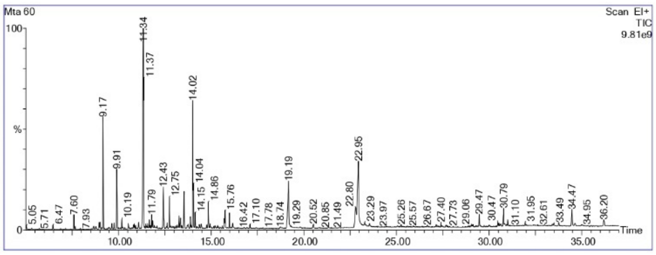

#	RT	Scan	Height	Area	Area %	Norm %
1	7.600	390	711,093,184	17,705,144.0	0.647	3.04
2	9.174	626	5,449,863,168	151,327,888.0	5.531	25.94
3	9.914	737	2,908,932,608	104,937,456.0	3.836	17.99
4	11.341	951	9,744,753,664	583,264,576.0	21.320	100.00
5	12.428	1114	2,021,084,800	91,518,512.0	3.345	15.69
6	12.755	1163	1,519,358,976	39,229,988.0	1.434	6.73
7	13.355	1253	478,220,128	17,217,744.0	0.629	2.95
8	13.542	1281	1,802,203,648	51,058,680.0	1.866	8.75
9	14.015	1352	6,195,302,912	216,761,648.0	7.923	37.16
10	14.149	1372	750,309,824	19,162,626.0	0.700	3.29
11	14.856	1478	1,266,672,640	39,668,012.0	1.450	6.80
12	15.756	1613	864,946,752	22,954,828.0	0.839	3.94
13	15.996	1649	749,675,584	17,667,480.0	0.646	3.03
14	19.190	2128	2,297,567,488	193,892,864.0	7.087	33.24
15	22.798	2669	944,065,024	79,408,792.0	2.903	13.61
16	22.951	2692	3,183,940,608	271,763,168.0	9.934	46.59
17	29.467	3669	589,354,624	16,197,070.0	0.592	2.78
18	30.787	3867	842,989,056	33,074,264.0	1.209	5.67
19	34.468	4419	805,143,616	40,335,108.0	1.474	6.92
20	36.195	4678	310,599,392	20,275,154.0	0.741	3.48

Inst() ACQUISITION PARAMETERS
, Inj=°C, Volume=0 µL, Split=:1, Carrier Gas=, Solvent Delay=5.00 min, Transfer Temp=210°C, Source Temp=210°C, Scan: 30 to 550Da, Column

Figure S14. Chromatograms and Quality Report retrieved from de GC-MS analysis with the peaks corresponding to the most abundant compounds for the seeds under the treatment 10 + UAE (**Table 1**).

Qualitative Report

File: C:\TurboMass\Biotec.PRO\Data\Mta 61.raw
Acquired: 09-Feb-22 07:49:04 PM
Description:
GC/MS Method: GC: MS: Vainilla.EXP
Sample ID: Mta 11

Printed: 16-Feb-22 11:45 AM
Page 1 of 1
Vial Number: 12

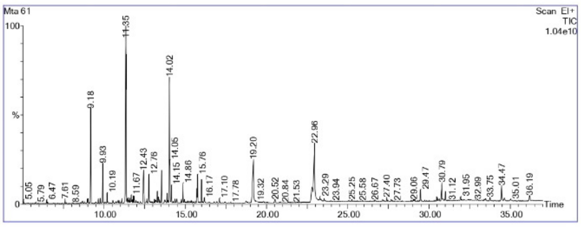

#	RT	Scan	Height	Area	Area %	Norm %
1	9.180	627	5,535,678,976	152,260,256.0	5.365	26.20
2	9.927	739	2,331,682,816	83,292,112.0	2.935	14.33
3	11.348	952	10,319,016,960	581,214,464.0	20.479	100.00
4	12.435	1115	1,912,932,864	87,662,712.0	3.089	15.08
5	12.762	1164	1,627,699,456	39,853,364.0	1.404	6.86
6	13.282	1242	635,794,752	18,325,454.0	0.646	3.15
7	13.542	1281	1,895,460,864	53,093,584.0	1.871	9.13
8	14.022	1353	7,284,275,712	258,843,600.0	9.120	44.53
9	14.149	1372	1,001,026,240	24,382,880.0	0.859	4.20
10	14.862	1479	1,111,351,552	35,251,592.0	1.242	6.07
11	15.709	1606	826,377,408	22,192,848.0	0.782	3.82
12	15.756	1613	1,632,924,416	42,966,372.0	1.514	7.39
13	15.996	1649	1,349,222,144	32,331,598.0	1.139	5.56
14	19.197	2129	2,531,985,152	215,159,600.0	7.581	37.02
15	22.798	2669	818,900,992	70,228,176.0	2.474	12.08
16	22.958	2693	3,327,857,152	263,116,736.0	9.271	45.27
17	29.473	3670	672,647,808	18,358,968.0	0.647	3.16
18	30.787	3867	1,050,480,640	39,091,484.0	1.377	6.73
19	34.468	4419	878,522,688	46,335,792.0	1.633	7.97
20	36.189	4677	311,836,992	19,376,524.0	0.683	3.33

Inst() ACQUISITION PARAMETERS
, Inj=°C, Volume=0 µL, Split=:1, Carrier Gas=, Solvent Delay=5.00 min, Transfer Temp=210°C, Source Temp=210°C, Scan: 30 to 550Da, Column

Figure S15. Chromatograms and Quality Report retrieved from de GC-MS analysis with the peaks corresponding to the most abundant compounds for the seeds under the treatment 11 + UAE (**Table 1**).

Qualitative Report

File: C:\TurboMass\Biotec.PRO\Data\Mta 62.raw
Acquired: 09-Feb-22 08:30:20 PM
Description:
GC/MS Method: GC: MS: Vainilla.EXP
Sample ID: Mta 12

Printed: 16-Feb-22 10:55 AM
Page 1 of 1
Vial Number: 13

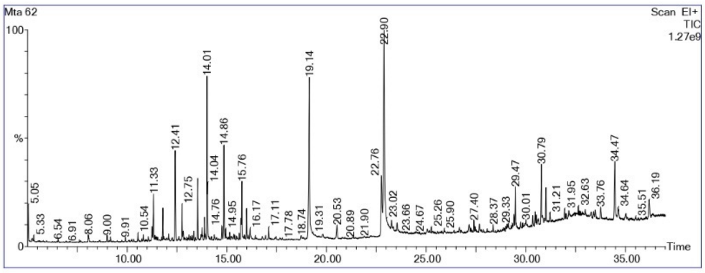

#	RT	Scan	Height	Area	Area %	Norm %
1	11.328	949	269,015,872	8,340,110.0	1.376	8.40
2	11.795	1019	190,245,632	4,505,814.5	0.744	4.54
3	12.415	1112	530,245,152	17,733,930.0	2.926	17.87
4	12.755	1163	209,790,624	5,420,923.0	0.895	5.46
5	13.542	1281	363,747,520	9,120,420.0	1.505	9.19
6	14.009	1351	953,894,080	30,249,642.0	4.991	30.48
7	14.862	1479	549,259,392	15,304,754.0	2.525	15.42
8	15.762	1614	337,355,968	9,290,078.0	1.533	9.36
9	19.144	2121	952,047,232	66,333,176.0	10.946	66.84
10	20.531	2329	81,148,488	4,628,938.0	0.764	4.66
11	22.765	2664	358,064,576	22,659,298.0	3.739	22.83
12	22.898	2684	1,216,754,560	99,243,168.0	16.376	100.00
13	23.265	2739	95,855,872	7,086,661.0	1.169	7.14
14	23.351	2752	57,841,916	5,394,442.5	0.890	5.44
15	23.545	2781	78,657,296	10,209,764.0	1.685	10.29
16	29.473	3670	251,900,976	7,354,370.0	1.214	7.41
17	30.787	3867	346,290,688	13,124,243.0	2.166	13.22
18	31.014	3901	201,725,008	6,069,687.0	1.002	6.12
19	34.468	4419	336,921,248	16,872,454.0	2.784	17.00
20	36.189	4677	108,369,976	6,877,347.0	1.135	6.93

Inst() ACQUISITION PARAMETERS
, Inj=°C, Volume=0 µL, Split=:1, Carrier Gas=, Solvent Delay=5.00 min, Transfer Temp=210°C, Source Temp=210°C, Scan: 30 to 550Da, Column

Figure S16. Chromatograms and Quality Report retrieved from de GC-MS analysis with the peaks corresponding to the most abundant compounds for the seeds under the treatment 12 + UAE (**Table 1**).

Qualitative Report

File: C:\TurboMass\Biotec.PRO\Data\Mta 63.raw
Acquired: 09-Feb-22 09:11:38 PM
Printed: 16-Feb-22 12:00 PM
Description:
GC/MS Method: GC: MS: Vainilla.EXP
Page 1 of 1
Sample ID: Mta 13
Vial Number: 14

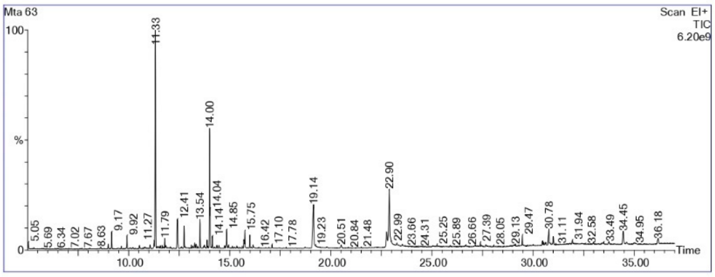

#	RT	Scan	Height	Area	Area %	Norm %
1	9.167	625	477,931,776	12,008,022.0	1.123	7.46
2	9.921	738	373,205,376	10,351,248.0	0.968	6.43
3	11.328	949	6,141,977,088	160,970,960.0	15.053	100.00
4	12.415	1112	826,913,600	38,921,728.0	3.640	24.18
5	12.748	1162	630,605,696	16,288,810.0	1.523	10.12
6	13.535	1280	812,673,472	20,808,446.0	1.946	12.93
7	14.002	1350	3,370,519,040	108,664,744.0	10.162	67.51
8	14.142	1371	334,474,528	7,616,909.0	0.712	4.73
9	14.849	1477	511,591,200	15,402,160.0	1.440	9.57
10	15.749	1612	485,847,200	12,579,454.0	1.176	7.81
11	15.989	1648	369,648,896	8,665,077.0	0.810	5.38
12	19.143	2121	1,234,663,808	89,231,360.0	8.344	55.43
13	22.758	2663	441,603,712	30,155,752.0	2.820	18.73
14	22.898	2684	1,645,327,872	131,069,736.0	12.257	81.42
15	23.265	2739	120,949,656	9,197,628.0	0.860	5.71
16	23.538	2780	80,802,864	8,142,737.5	0.761	5.06
17	29.467	3669	309,539,552	8,942,640.0	0.836	5.56
18	30.780	3866	452,237,472	18,182,716.0	1.700	11.30
19	34.455	4417	363,098,848	18,587,668.0	1.738	11.55
20	36.182	4676	155,124,800	9,342,043.0	0.874	5.80

Inst() ACQUISITION PARAMETERS
, Inj=°C, Volume=0 µL, Split=:1, Carrier Gas=, Solvent Delay=5.00 min, Transfer Temp=210°C, Source Temp=210°C, Scan: 30 to 550Da, Column

Figure S17. Chromatograms and Quality Report retrieved from de GC-MS analysis with the peaks corresponding to the most abundant compounds for the seeds under the treatment 13 + UAE (**Table 1**).
